# Differential responses of mineral-associated organic carbon and particulate organic carbon upon desertification of the Inner Mongolian grassland

**DOI:** 10.3389/fmicb.2026.1814844

**Published:** 2026-05-07

**Authors:** Hao Peng, Baozhu Dong, Gaowa Naren, Lijuan Ma, Xiaodong Yu

**Affiliations:** 1 College of Resources and Environmental Economics, Inner Mongolia University of Finance and Economics, Hohhot, China; 2 School of Life Sciences, Inner Mongolia University, Hohhot, China; 3 College of Resources and Environmental Sciences, Inner Mongolia Agricultural University, Hohhot, China

**Keywords:** desert steppe, mineral-associated organic carbon, particulate organic carbon, soil microorganism, soil organic carbon, typical steppe

## Abstract

**Introduction:**

Grassland desertification substantially reduces soil organic carbon (SOC) stocks. However, the responses of two distinct SOC fractions—mineral-associated organic carbon (MAOC) and particulate organic carbon (POC)—to desertification, as well as the underlying mechanisms governing these responses, remain poorly understood.

**Methods:**

To address this knowledge gap, we conducted a field study across the typical steppe (TS) and desert steppe (DS) ecosystems of Inner Mongolia, China, to investigate the dynamics of MAOC and POC and their underlying drivers, including iron/aluminum (Fe/Al) oxides, exchangeable calcium (Ca_*ex*_) and microbial community composition.

**Results:**

Our results reveal a significant decline in MAOC concentrations under desertification, with mean values of 15.66 g/kg (0–10 cm) and 14.99 g/kg (10–20 cm) in the TS, and 12.10 g/kg (0–10 cm) and 11.64 g/kg (10–20 cm) in the DS. Unlike MAOC, POC concentrations significantly varied in only the surface layers of TS and DS, with mean values of 6.22 g/kg in the TS and 4.73 g/kg in the DS. This indicates divergent vertical responses of MAOC and POC to grassland desertification. In the DS, reduced fungal abundance—coupled with lower concentrations of amorphous Fe/Al oxides (Fe_*o*_/Al_*o*_) in the fine soil fraction—collectively constrained MAOC accumulation. However, because aboveground biomass contributes the majority of litter input preferentially to the surface soil layer, sparse vegetation in the DS primarily limits POC accumulation in the surface horizon.

**Discussion:**

This study provides the first comprehensive assessment of the differential responses of MAOC and POC to desertification in Inner Mongolian grasslands and elucidates the associated biogeochemical mechanisms.

## 1 Introduction

Grassland ecosystems cover an area of 52.5 million km^2^ and store ∼34% of the terrestrial carbon stock, thereby playing a vital role in soil carbon sequestration (White et al., 2000; Bardgett et al., 2021). Even a slight reduction in grassland soil organic carbon (SOC) can trigger significant changes in atmospheric CO_2_ levels (Ochoa-Hueso et al., 2018; Xue et al., 2023). Therefore, understanding SOC sequestration and its stability in grassland is essential for predicting the feedback of grassland carbon dynamics to climate changes (Bai and Cotrufo, 2022). Unfortunately, grasslands worldwide have experienced significant declines in ecosystem functions, with ongoing desertification progressively intensifying reductions in SOC storage (Eze et al., 2018; Yang et al., 2019). The SOC content decreased significantly with the progression of grassland desertification, by 7.1% in areas experiencing light desertification, 37.7% in those undergoing severe desertification, and 48.2% in those subjected to very severe desertification (An et al., 2019). Numerous studies have quantified the negative impact of desertification on SOC (Allington and Valone, 2010; Lu et al., 2014; Tang et al., 2015; An et al., 2019). However, the understanding of how different fractions of SOC respond to grassland desertification is limited.

SOC is generally divided into two fractions: mineral-associated organic carbon (MAOC) and particulate organic carbon (POC). MAOC and POC differ in their formation processes as well as their physical and chemical properties, which fundamentally influence their residence times in soil—less than 10 years for POC, whereas MAOC can persist in soil for decades to centuries (Lavallee et al., 2020; Cotrufo and Lavallee, 2022). POC originates from the fragmentation of plant and microbial residues, consisting primarily of large polymers. In contrast, MAOC is formed from low-molecular-weight compounds that are either leached from plant residues or exuded by plant roots. These compounds are associated with minerals directly or following microbial assimilation and are subsequent released as microbial necromass (Lavallee et al., 2020; Cotrufo and Lavallee, 2022). The final storage of MAOC or POC is dependent not only on the input of these organic materials but also on the fixation conditions of the soil. In recent years, the protective roles of soil aggregates and soil minerals have been widely recognized as key mechanisms underlying the persistence of SOC (Schmidt et al., 2011; Dungait et al., 2012). POC, which is predominantly of plant origin, persists in soil through inherent biochemical recalcitrance, physical protection in aggregates and/or microbial inhibition (Golchin et al., 1994). Soils with higher contents of iron and aluminum oxides have a stronger adsorption capacity for MAOC, mainly through ligand exchange and electrostatic interactions, to adsorb soil organic matter (Saidy et al., 2013).

The drought threshold represents a critical factor governing the accumulation of MAOC and POC in grassland ecosystems. The contents of MAOC and POC follow the descending order: meadow steppe (MS) > typical steppe (TS) > desert steppe (DS) (Chen et al., 2025). Relative to DS, MS and TS receive higher rainfall intensity and experience more humid climatic conditions. Increased precipitation enhances vegetation biomass production and subsequent carbon input into soils, thereby promoting POC formation and facilitating the downward leaching of MAOC into deeper soil layers (Chen et al., 2020; Wang et al., 2022). Soil moisture plays a pivotal role in regulating the distribution and stabilization patterns of POC and MAOC. Elevated moisture levels stimulate plant-derived carbon inputs, thus enhancing POC accumulation. Conversely, under high-moisture conditions, reduced soil redox potential induces the reductive dissolution of iron and aluminum oxides—processes that impair the capacity of mineral surfaces to stabilize MAOC (Angst et al., 2021; Liu et al., 2026). In arid regions, MAOC constitutes the dominant fraction of soil organic carbon (Díaz-Martínez et al., 2024), primarily because carbon-poor soils typically exhibit low carbon inputs and limited suppression of microbial activity, rendering protected MAOC comparatively more persistent than labile POC (Lugato et al., 2021). In desert steppe, sparse vegetation cover renders the soil highly susceptible to wind erosion, accelerating the loss of fine particles and resulting in the highest proportion of sand fractions in desert steppe (Yan et al., 2013). Generally, clay particles—compared with coarser fractions—offer a substantially greater specific surface area and a higher density of reactive sites for adsorbing organic carbon (Christensen, 2001; Lützow et al., 2007). Consequently, the depletion of fine particles in desert steppe significantly constrains MAOC accumulation.

In China, approximately 70% of grasslands are experiencing varying levels of degradation, causing a substantial decrease in the SOC stock (Barthold et al., 2013). However, the responses of two distinct fractions of SOC to grassland desertification, as well as the underlying mechanisms governing these responses, remain poorly understood. This limitation precludes reliable predictions of soil carbon stock dynamics. To address these gaps, we conducted an experiment in both the typical steppe (TS) and desert steppe (DS) ecosystems of Inner Mongolia to investigate the dynamics of MAOC and POC, as well as their underlying driving factors. The following key questions will be addressed. (1) What are the differences in the distribution patterns of MAOC and POC between the TS and DS in Inner Mongolia? (2) How are exchangeable calcium (Ca_*ex*_) and iron/aluminum (Fe/Al) oxides distributed between fine (<53 μm) and coarse (>53 μm) soil fractions in the TS and DS ecosystems? Which metal ions exhibit a distribution pattern similar to that of MAOC and/or POC? (3) What are the differences in microbial community composition between TS and DS soils, and how are these differences related to the distribution patterns of MAOC and POC? This study provides the first comprehensive analysis of the differential responses of MAOC and POC to desertification in the Inner Mongolian grassland and their underlying mechanisms.

## 2 Materials and methods

### 2.1 Study area

**FIGURE 1:**
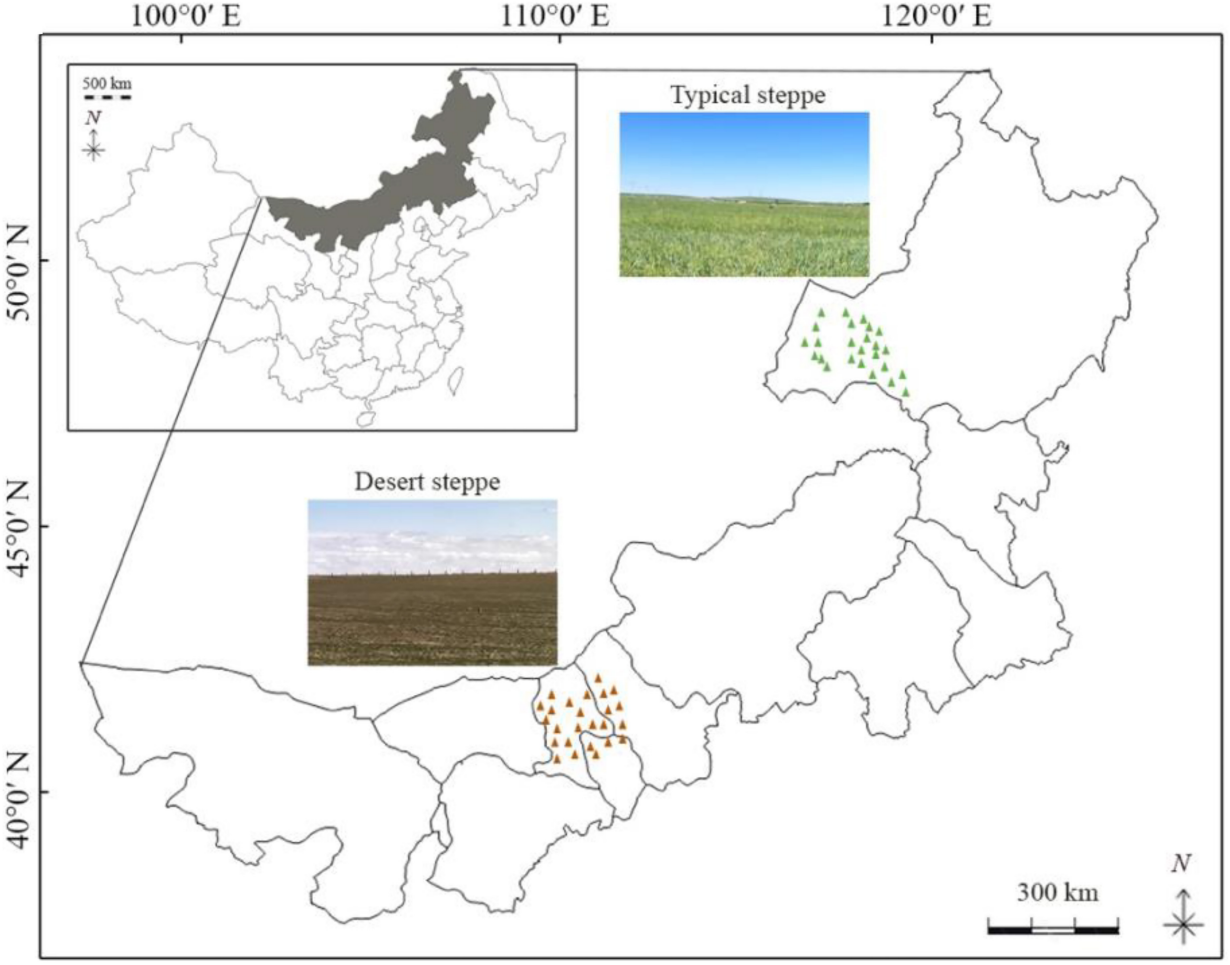
Spatial distribution of sampling sites across different grassland types. Green and brown triangles denote sampling locations in TS and DS, respectively.

Grasslands in Inner Mongolia account for the main part of the temperate grasslands across the isohyet of 200 mm and 400 mm, and they are principally classified as meadow steppe (350–500 mm), typical steppe (300–400 mm) and desert steppe (≤ 200 mm) based on climatic conditions and the Chinese vegetation classification system. Overgrazing has been one of the most significant causes of grassland degradation in recent decades (Wang and Zhu, 2001; Long et al., 2005). The sampling area of this study was located in the DS and TS of Inner Mongolia, China. The sampling period was from August 21st to 23rd, 2023. The soil samples were collected from Siziwang Banner in Ulanqab City, Darhan Muminggan United Banner in Baotou City, and Wuchuan County in Hohhot City in DS, and from Xin Barag Left Banner and Xin Barag Right Banner of Hulun Buir City in TS. The community biodiversity in the DS of Inner Mongolia is predominantly characterized by *Stipa klemenzii* and *Stipa breviflora*. In contrast, the community diversity in TS is dominated by species such as *Leymus chinensis, Stipa grandis*, and *Cleistogenes squarrosa* (Ma et al., 2006). According to the “China Surface Meteorological Observation Historical Dataset” of the National Meteorological Science Data Center,^1^ in 2023, the precipitation in DS area was 210.2 mm and the annual average temperature was 2.44°C; in TS, the average annual temperature in this area is 1.5°C, with an average annual precipitation of 255.9 mm. The soil in DS is mainly brown calcareous soil, while in TS, it is black calcareous soil and chestnut calcareous soil (Bai et al., 2008). The locations of the sampling points were shown in Figure 1 and Supplementary Table 1.

### 2.2 Soil sampling

In August, 2023, 25 sampling points were established in each of these two steppes. Each plot consisted of a 10 × 10 m quadrat, and the average inter-plot distance exceeded 20 km. The S-shaped transection method was employed, and three soil cores were taken from the 0 to 20 cm depth in each quadrat and split into two depths (0–10 and 10–20 cm). Soils from the same depth were mixed in situ for each quadrat as one composite sample and each quadrat served as a replicate for analysis, given the large heterogeneity of SOC composition and microenvironments experienced by soil microbes. After collection, the soil samples were divided into two portions. One portion were kept on ice and sent back to the laboratory where they were stored in a -80 °C freezer for soil DNA extraction and metagenomic sequencing. We randomly selected 6 out of 25 replicates at each steppe type for metagenome analysis. The other portion was air-dried and subsequently sieved through a 2-mm mesh to remove plant litter and root fragments prior to analysis of soil physicochemical properties.

### 2.3 Soil organic carbon fraction separation and content determination

A particle size fractionation method was used to separate SOC into MAOC and POC (Cambardella and Elliott, 1992). The main steps of the particle size fractionation procedure were as follows: 30 g of air-dried, 2-mm sieved soil and 100 mL of sodium hexametaphosphate solution (NaPO_3_)_6_ (5 g/L) were added to conical flask. The mixture was shaken on an orbital shaker for 18 h (25 °C, 180 r/min), after which the suspension was passed through a 53 μm nylon sieve and rinsed with distilled water until the effluent became clear. The fine fraction (<53 μm) was used for MAOC analysis, whereas the coarse fraction (>53 μm) was analyzed for POC concentration. After separation, both the fine and coarse fractions were dried at 60°C, weighed, and analyzed for organic carbon and metal ions (Ca, Fe, and Al), respectively.

The organic carbon content in each fraction was determined using the potassium dichromate-external heating method (Sokol et al., 2019). MAOC and POC were calculated according to Equations (1) and (2), with units expressed in g/kg.


$$MAOC=\left(\Delta M1/M\right)\times C{}_{M\,A\,O\,C}$$ (1)

$$POC=\left(\Delta M2/M\right)\times C{}_{P\,O\,C}$$ (2)

Where ΔM1 represents the oven-dry weight of the fine fraction after separation (g); ΔM2 denotes the oven-dry weight of the coarse fraction after separation (g); M is the total mass of the soil sample before separation (g); C_*MAOC*_ indicates the concentration of organic carbon from the fine soil determined by the potassium dichromate-external heating method (g/kg); and C_*POC*_ refers to the organic carbon concentration of the coarse soil determined by the potassium dichromate-external heating method (g/kg).

### 2.4 Soil properties measurement

Soil pH was measured in a 2.5:1 water-soil mixture using a pH meter (FiveEasy Plus, Mettler-Toledo, Switzerland). Soil moisture was assessed via the oven-drying method, where fresh soil was dried at 102 °C until a constant weight was achieved. In parallel with soil sampling with corer, we measured soil temperature at 5 cm (medium of 0–10 cm) and 15 cm (medium of 10–20 cm) of the soil column with WET-2 sensor (Delta-T Devices, United Kingdom). The vegetation coverage was determined by comparing the soil area covered by plants to the exposed ground in the sample plot (10 m × 10 m).

### 2.5 Determination of free iron and aluminum oxide (Fe_*d*_ and Al_*d*_) in soil

A 1.0 g soil sample, previously passed through a 0.25 mm sieve, was added to a 100 mL centrifuge tube pre-filled with 2.5 mL of 1 mol/L sodium bicarbonate (NaHCO_3_) and 20 mL of 0.3 mol/L sodium citrate (C_6_H_5_Na_3_O_7_) solution. The mixture was heated in a water bath at 85°C, then 0.5g sodium Dithionite (Na_2_O_4_S_2_) was added, and the mixture was stirred continuously with a glass rod for 15 min. The sample was centrifuged at 4,000 rpm for 10 min. The supernatant was then transferred to a 100 mL flask. The pellet was washed twice with 1 mol/L sodium chloride (NaCl) solution; following each wash and subsequent centrifugation, the resulting supernatant was also transferred to the same flask. The mineral element content of the test solution was determined by ICP-OES (Avio 200, PerkinElmer, United States).

### 2.6 Determination of amorphous iron and aluminum oxides (Fe_*o*_ and Al_*o*_) in soil

A 1.0 g soil sample that has passed through a 0.25 mm sieve was transferred into a conical flask, followed by the addition of 50 mL oxalic acid-ammonium oxalate [H_2_C_2_O_4_–(NH_4_)2C_2_O_4_] solution. The mixture was then shaken for 2 h on a mechanical shaker and subsequently filtered. The entire experimental process should be carried out under light-shielded conditions. The mineral element content of the test solution was determined by ICP-OES (Avio 200, PerkinElmer, United States).

### 2.7 Determination of exchangeable calcium (Ca_*ex*_) in soil

The content of Ca_*ex*_ was determined by atomic absorption spectrometry following extraction with 1 mol/L ammonium acetate (NH_4_OAc). A 2.0 g soil sample (<2 mm) was subjected to repeated extraction with 20 mL of 1 mol/L NH_4_OAc to obtain a final volume of approximately 100 mL. After filtration and adjusting the extract to a final volume of 100 mL, the concentrations of Ca_*ex*_ was determined using flame atomic absorption spectroscopy (ZA3000, Hitachi, Japan).

### 2.8 Soil DNA extraction, library construction and metagenomic sequencing

Soil DNA extraction and subsequent metagenomic sequencing analysis in this study were performed by Sangon Biotech (Shanghai) Co., Ltd. A 200-mg aliquot of each soil sample was subjected to pretreatment, followed by total DNA extraction using the Mag-Bind Soil DNA Kit (Omega, M5635-02, United States). If the DNA quality met the library construction standards (the main band of the sample DNA electrophoresis was clear or only slightly degraded), 500 ng was taken from each DNA sample for library construction. The Hieff NGS^®^MaxUp II Illumina^®^DNA Library Preparation Kit (12200ES96, YEASEN, China) was used to construct the sequencing library, and the library was sequenced by the NovaSeq 6000 sequencer (Illumina, United States) for paired-end sequencing.

### 2.9 Metagenomic sequencing data analysis, gene assembly, gene function and species annotation

Low-quality sequences and short sequences were removed (Fastp 0.36), and the high-quality sequences obtained were subjected to subsequent analysis. The quality-controlled sequences were assembled (Megahit 3.13), and the open reading frames of the contigs obtained after assembly were predicted (Prodigal 2.60). Genes with DNA sequence lengths over 100 bp were considered as the candidate gene set. Redundancy analysis was performed on the gene set, and the abundance of each gene was calculated. The amino acid sequences of the gene set were compared with the NCBI protein database (NCBI non-redundant protein sequences) using DIAMOND to obtain functional annotations and homologous species information. Based on the NCBI microbial classification database, the species classification annotation information of the genes was analyzed, and the relative abundance of species was statistically analyzed at different taxonomic levels, including Kingdom, Phylum, Class, Order, Family, Genus and Species.

### 2.10 Data analysis

Data organization was performed using Microsoft Excel 2023. Box plots were generated using Origin Pro 2021software. All statistical analyses were conducted using SPSS version 20.0. One-way ANOVA was employed to assess differences in soil physicochemical properties, MAOC, POC and SOC between the DS and TS. A paired *t*-test was used to evaluate differences in soil physicochemical properties, MAOC, POC and SOC between the surface (0–10 cm) and subsurface (10–20 cm) soil layers. Redundancy analysis (RDA) was performed using CANOCO 5 (Microcomputer Power, Ithaca, NY, United States) to evaluate the influence of environmental variables—including VC, pH, ST, SM, Fe/Al oxides, Ca_ex_—on the concentrations of MAOC and POC. Soil physicochemical properties were designated as explanatory variables.

## 3 Results

### 3.1 Concentrations of both MAOC and POC in the DS were lower than those in the TS

**FIGURE 2:**
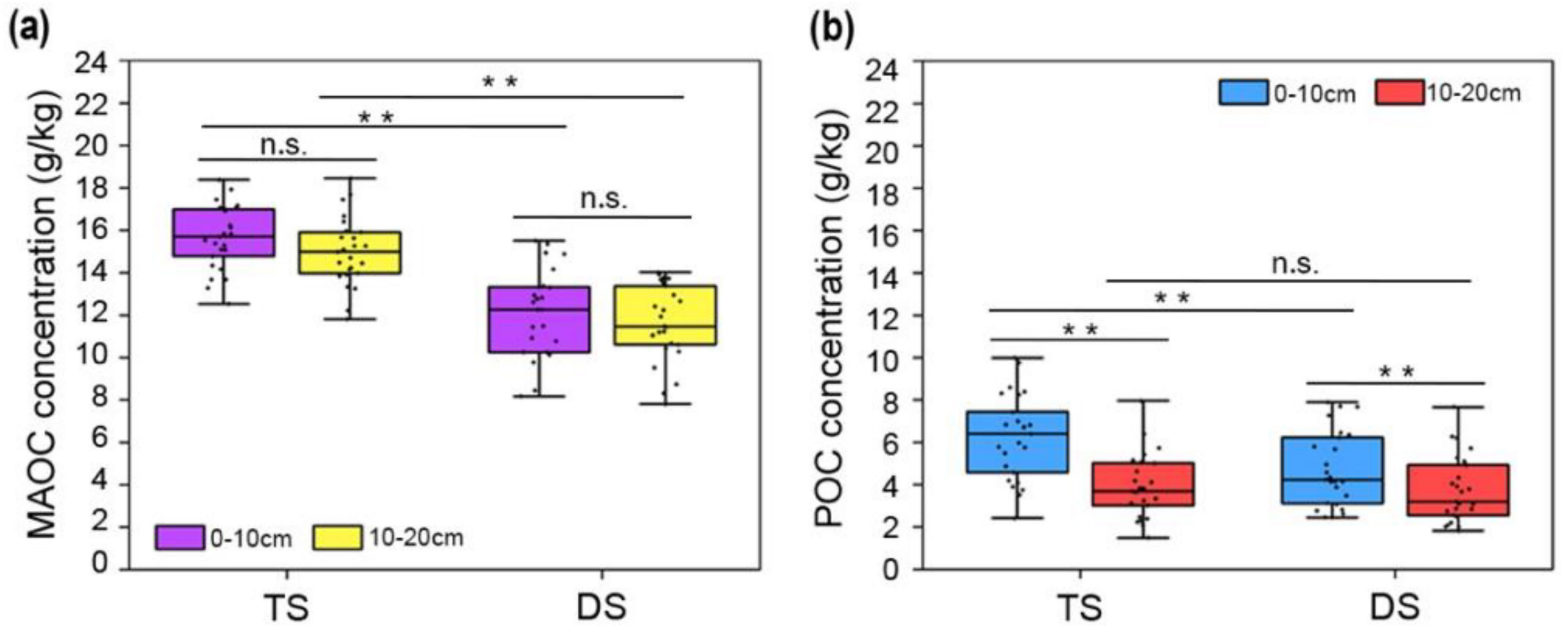
The concentrations of MAOC and POC in the different steppes. **(a)**, The concentrations of MAOC in the surface (0–10 cm) and subsurface (10–20 cm) layers of TS and DS. **(b),** The concentrations of POC in the surface (0–10 cm) and subsurface (10–20 cm) layers of TS and DS. One-way ANOVA was employed to conduct statistical analyses between different steppes; paired *t*-tests were used to compare means across different soil layers. Asterisks denote significant differences (**P < 0.01). “n.s.” means no significant difference. TS, typical steppe; DS, desert steppe.

In this study, we report that the concentrations of MAOC in the fine fraction were essentially greater than those of POC in the coarse pool in both the DS and TS of the Inner Mongolia grassland (Figure 2). Significant differences in MAOC concentration were detected between these two steppe types, with mean values of 15.66 g/kg (0–10 cm) and 14.99 g/kg (10–20 cm) in the TS, and 12.10 g/kg (0–10 cm) and 11.64 g/kg (10–20 cm) in the DS. No statistically significant differences were observed between the surface and subsurface layers within each steppe (Figure 2a). Unlike MAOC, POC concentrations significantly varied in only the surface layers of the TS and DS, with mean values of 6.22 g/kg in TS and 4.73 g/kg in DS. Although the concentrations of POC in subsurface layers were comparable between the TS (3.92 g/kg) and DS (3.76 g/kg), they were significantly lower than those in the surface layers within the same type of steppe (Figure 2b). On the basis of the above data, compared to the coarse fractions, the fine fraction presented a higher organic carbon density in both steppe types. Compared with that in the subsurface layer, the POC in the surface layer was more sensitive to grassland degradation, whereas the MAOC in both soil layers was markedly reduced in the DS.

### 3.2 The SOC content in the surface soil layer was significantly decreased in the DS

**FIGURE 3:**
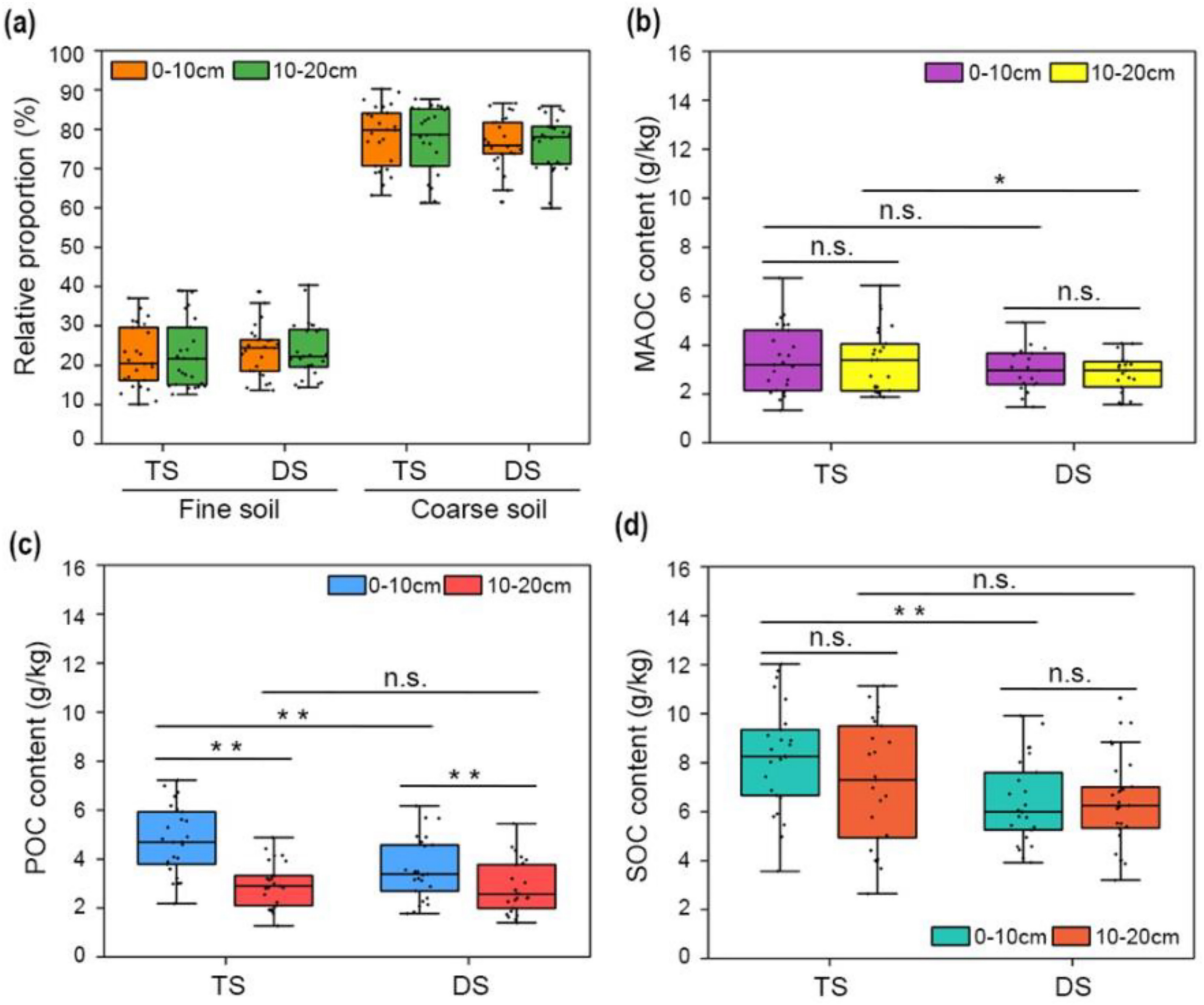
Particle-size distribution and contents of MAOC, POC, and SOC in TS and DS. **(a)**, The relative proportions of fine (<53 μm) and coarse (>53 μm) soil particles in TS and DS. The contents of MAOC **(b)**, POC **(c)** and SOC **(d)** in the surface (0–10 cm) and subsurface (10–20 cm) layers of TS and DS. One-way ANOVA was employed to conduct statistical analyses among different steppes; paired *t*-tests were used to compare means across different soil layers. Asterisks denote significant differences (*P < 0.05, **P < 0.01). “n.s.” means no significant difference. TS, typical steppe; DS, desert steppe.

Even the concentrations of MAOC were significantly greater than those in coarse particles (Figure 2), the final content of MAOC in the whole soil bulk was also affected by the proportion of fine particles within the entire soil matrix. The percentages of soil particles with a size less than 53 μm were markedly lower than those of larger-sized particles, with a ratio of approximately 1:4, which remained consistent across both the surface and subsurface layers of different grassland types (Figure 3a). Therefore, the lower proportion of fine soil particles decreased the MAOC content in the total soil, with mean values of 3.44 g/kg (0–10 cm) and 3.39 g/kg (10–20 cm) in the TS, and 2.81 g/kg (0–10 cm) and 2.71 g/kg (10–20 cm) in the DS (Figure 3b). In contrast, the proportions of POC in total SOC were improved. In the vertical direction, the POC contents in both grassland types significantly decreased with increasing soil depth, with mean values of 4.79 g/kg (0–10 cm) and 2.95 g/kg (10–20 cm) in the TS, and 3.58 g/kg (0–10 cm) and 2.82 g/kg (10–20 cm) in the DS. The POC content in the surface layer of the DS was significantly lower than that in the same layer of the TS, with no significant difference in POC observed in the subsurface layer between the two steppes (Figures 3b,c). As the sum of MAOC and POC, the SOC content decreased with the grassland desertification, with a significant difference observed in the surface layer compared with that in the TS (Figure 3d).

### 3.3 Exchangeable calcium (Ca_ex_) was preferentially associated with fine soil particles and accumulated more in the DS

**FIGURE 4:**
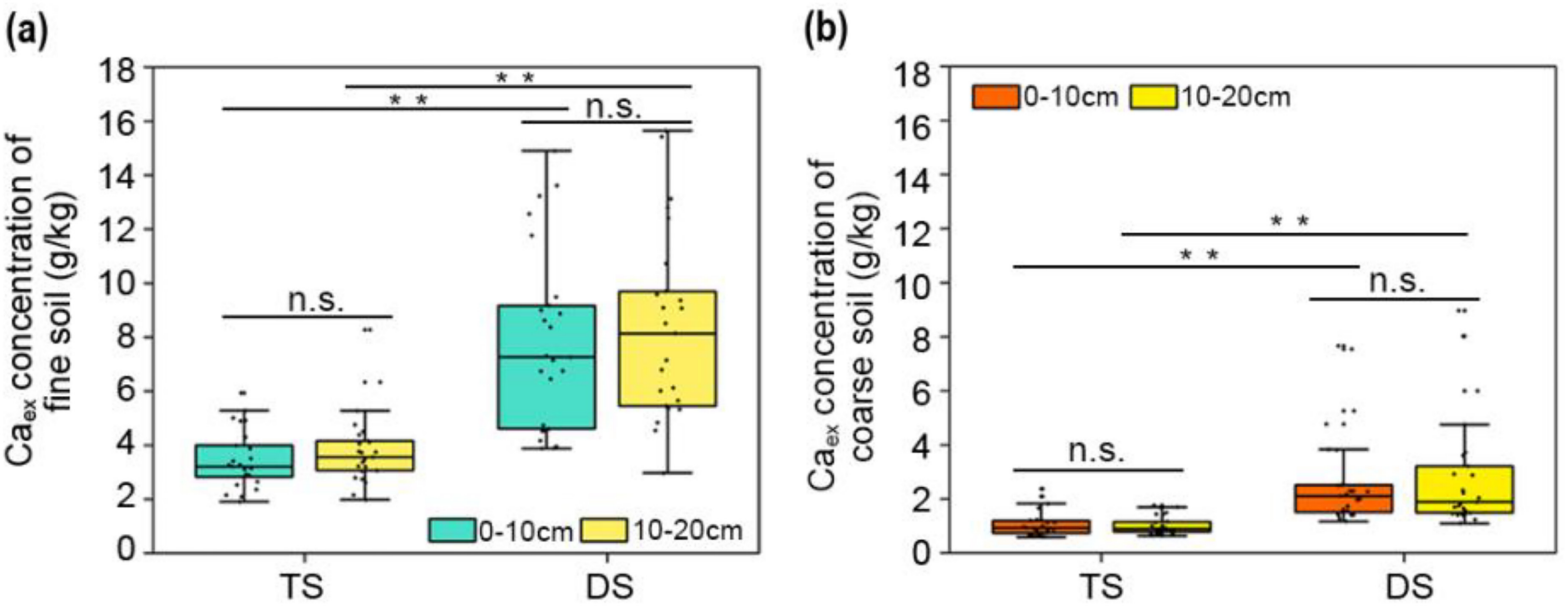
The concentrations of Ca_ex_ in the soils of TS and DS. **(a)**, The concentrations of Ca_ex_ in fine particles (<53 μm) of TS and DS. **(b)**, The concentrations of Ca_ex_ in coarse particles (>53 μm) of TS and DS. One-way ANOVA was employed to conduct statistical analyses among different steppes; paired *t*-tests were used to compare means across different soil layers. Asterisks denote significant differences (**P < 0.01). “n.s.” means no significant difference. TS, typical steppe; DS, desert steppe.

Consistent with the distribution pattern of MAOC, Ca_ex_ also tended to be associated with the fine soil particles rather than the coarse particles in both the TS and DS (Figure 4). However, in contrast to MAOC, the concentration of Ca_ex_ in the DS was significantly greater than that in the TS, regardless of whether it was in the fine or coarse soil fractions. In the case of the same type of particle within the same steppe, no statistically significant difference in Ca_ex_ concentration was observed between the 0–10 cm and 10–20 cm soil layers (Figure 4), indicating a uniform vertical distribution of Ca_ex_ in the soil profile. In summary, compared to coarse soil, fine particles have a greater capacity to Ca_ex_, and compared with those in the TS soil, the Ca_ex_ contents in both particle sizes in the DS soils were significantly greater.

### 3.4 Amorphous Fe/Al oxidate (Fe_o_/Al_o_) incubated in fine soil particles in the TS was greater than that in the DS

**FIGURE 5:**
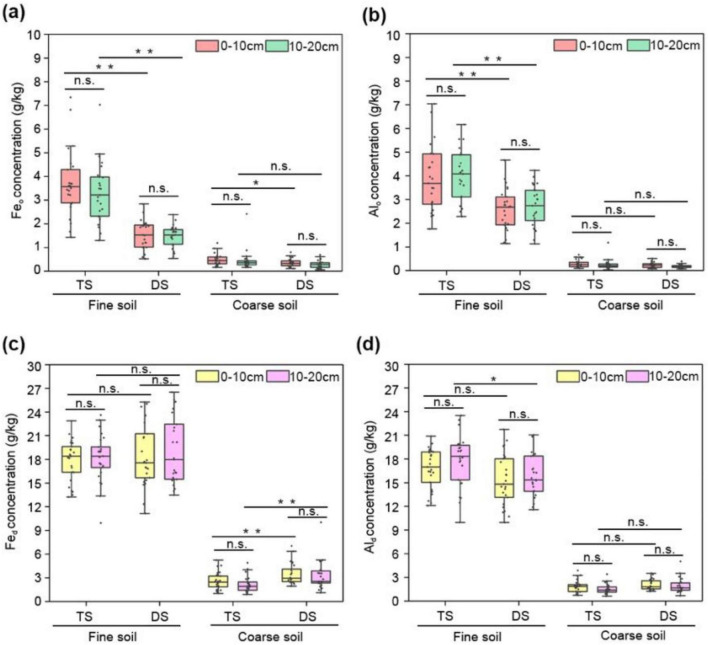
Distribution of Fe_o_/Al_o_ and Fe_d_/Al_d_ in different soil fractions of TS and DS. The concentrations of Fe_o_ **(a)**, Al_o_ **(b)**, Fe_d_ **(c)** and Al_d_ **(d)** in different soil fractions. One-way ANOVA was employed to conduct statistical analyses among different steppes; paired *t*-tests were used to compare means across different soil layers. Asterisks denote significant differences (*P < 0.05, **P < 0.01). “n.s.” means no significant difference. TS, typical steppe; DS, desert steppe.

To investigate the distributional relationship between Fe/Al minerals and different types of SOC, two forms of Al/Fe oxides in the bulk soil, amorphous Fe/Al oxides (Fe_o_/Al_o_) and free Fe/Al oxides (Fe_d_/Al_d_), were analyzed in this study. Both metallic oxides were predominantly embedded in the fine particles of these two types of steppes (Figure 5). There was no significant difference in the Fe_d_ of the fine fractions between the two steppes. However, the Fe_o_ concentration in these smaller particles were considerably higher in the TS than in the DS (Figures 5a,c). The Al_o_ distribution displayed a pattern analogous to that of Fe_o_, with significantly higher concentrations observed in the TS than in the DS (Figure 5b). For free Al oxides, significant differences in content between the TS and DS were observed exclusively in the 10–20 cm layer (Figure 5d). No significant differences between the surface and subsurface were observed for either Al_d_/Fe_d_ or Al_o_/Fe_o_ for both types of steppes (Figure 5). Therefore, the distribution of Fe/Al oxides between soil particles < 53 μm and > 53 μm closely paralleled that of MAOC, with higher concentrations of both Fe/Al oxides and MAOC colocalized in the fine soil fraction. Furthermore, the concentrations of Al_o_/Fe_o_ and MAOC in the TS were significantly greater than those in the DS, indicating a strong association between the distribution of Al_o_/Fe_o_ and MAOC in grassland soils.

### 3.5 The Differences in microbial community composition in soil between TS and DS

**FIGURE 6:**
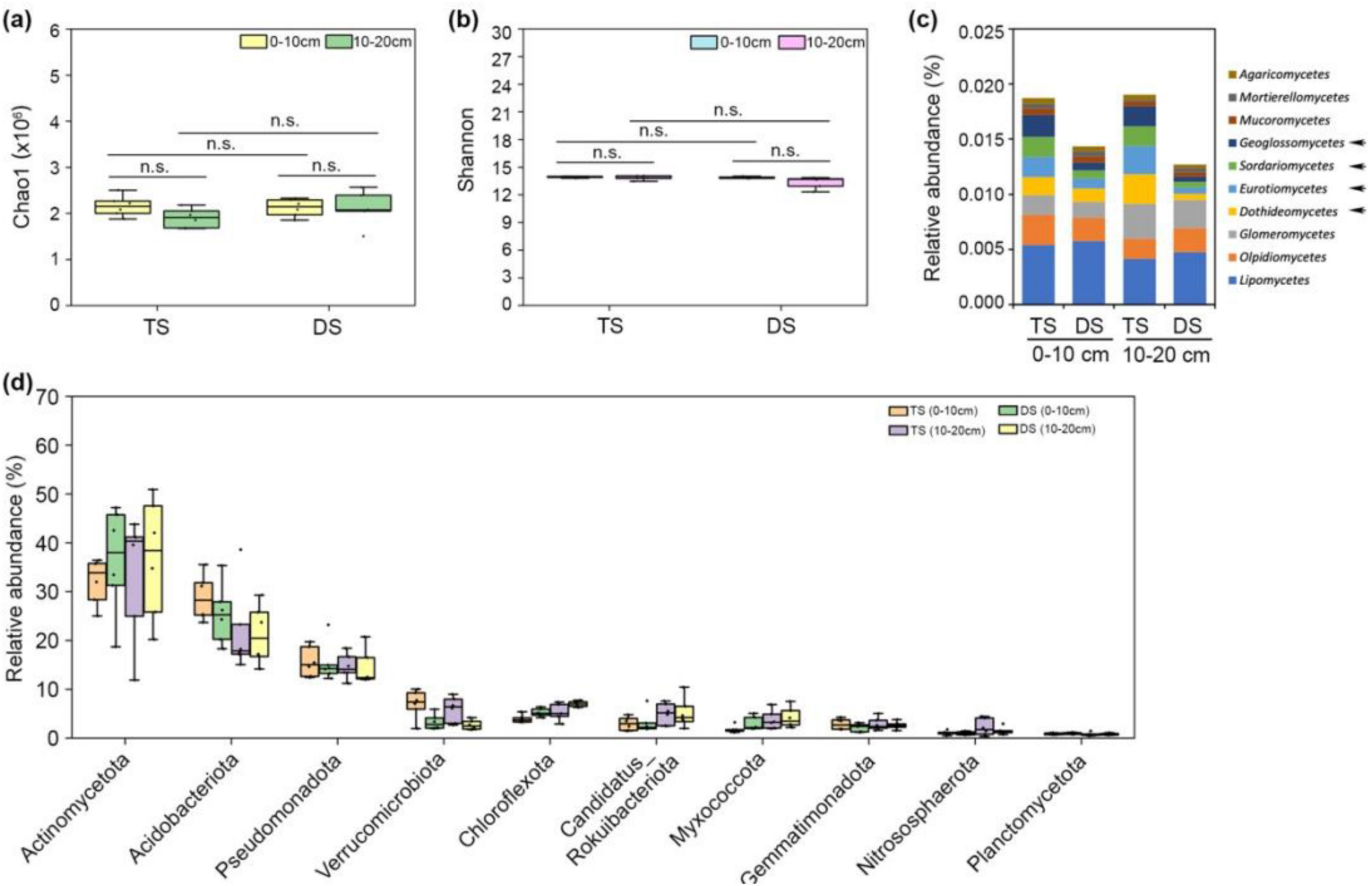
Microbial community composition in different steppe soils. Boxplot comparison of the Chao1 index **(a)** and Shannon index **(b)** for microbial communities across different steppe soil samples. **(c)**, The top 10 fungal classes in the 0–10 cm and 10–20 cm soil layers of TS and DS. Arrowheads indicate the fungal classes exhibiting substantial differences in relative abundance between TS and DS. **(d)**, The relative abundance of the top 10 bacterial phyla in TS and DS. One-way ANOVA was employed to conduct statistical analyses among different steppes; paired *t*-tests were used to compare means across different soil layers. “n.s.” means no significant difference. TS, typical steppe; DS, desert steppe.

Soil metal ions are commonly recognized as stabilizing factors that govern the quantity and persistence of SOC, whereas soil microorganisms serve as driving agents that mediate the input of plant-derived organic matter into the soil. Metagenomic sequencing was utilized to investigate the microbial community composition in soils from the TS and DS. The microorganisms were predominantly bacteria, accounting for approximately 98%, followed by archaea, viridiplantae, fungi, and others (Supplementary Table 2). The alpha-diversity analysis of the microbial communities revealed no significant differences in species abundance (Chao1 index) (Figure 6a) or species evenness (Shannon index) (Figure 6b) among the various steppe soil samples. Fungi serve a critical function in soil ecosystems, primarily through their essential contributions to decomposition and nutrient cycling. The fungal abundances in the DS were lower than those in the TS in both soil layers (Supplementary Table 2), indicating that the fungus-mediated transformation of plant-derived organic matter into soil organic carbon was less pronounced in the DS. A comparison of the list of the top 10 fungal classes from the TS and DS revealed that *Dothideomycetes*, *Eurotiomycetes*, *Sordariomycetes*, and *Geoglossomycetes* were more abundant in the TS in both soil layers (Figure 6c). Bacteria constituted the predominant component of soil microorganisms, and the top 10 phyla in the TS and DS are presented in Figure 6d. The most abundant bacterial phylum in both steppe soils was *Actinomycetota*, followed by *Acidobacteriota*, *Pseudomonadota*, and *Verrucomicrobiota*. Among these phyla, only *Verrucomicrobiota* consistently demonstrated a significantly greater abundance in the TS across both soil layers (Figure 6d). Members of the phylum *Verrucomicrobiota* play crucial roles in plant-microbe interactions, contributing to carbon cycling through the degradation of complex organic polymers such as cellulose, pectin, and starch (Naziêbło et al., 2026). The lower abundance of *Verrucomicrobiota* in the DS impairs its capacity for soil organic carbon accumulation. On the basis of the above data, the abundance and evenness of soil microbial species were comparable between the TS and DS. However, the composition of fungal and bacterial communities differed between the two steppes, with some microorganisms involved in soil carbon sequestration being more abundant in the TS than in the DS. This may partly account for the higher SOC content observed in the TS.

### 3.6 Relationships between the environmental factors and MAOC/POC

**FIGURE 7:**
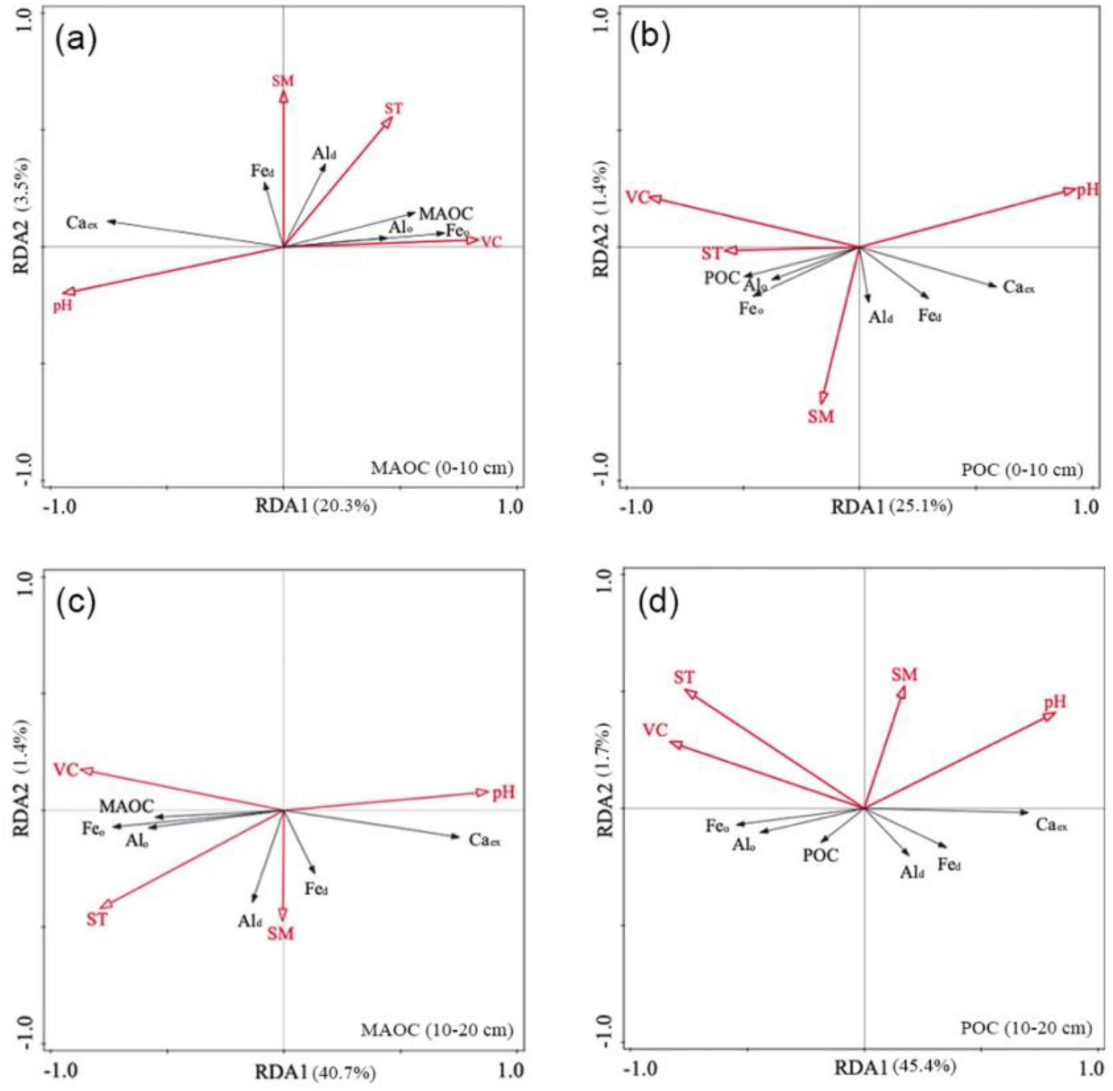
Redundancy analysis (RDA) of environmental variables and MAOC (0–10 cm) **(a)**, POC (0–10 cm) **(b)**, MAOC (10–20 cm) **(c)**, and POC (10–20 cm) **(d)**. MAOC, mineral-associated organic carbon; POC, particulate organic carbon; Ca_ex_, exchangeable calcium; Fe_o_, amorphous iron oxide; Fe_d_, free iron oxide; Al_o_, amorphous aluminum oxide; Al_d_, free aluminum oxide; SM, soil moisture; ST, soil temperature; VC, vegetation coverage.

To assess the effects of environmental factors and vegetation coverage on the concentrations of MAO/POC, as well as soil metal ions, across different grassland types, RDA—a multivariate statistical technique—was conducted. Climate showed an indirect effect on SOC by influencing soil physicochemical properties, including pH, Ca_ex_, and Al/Fe oxides. In both surface and subsurface soil layers, the angles between MAOC/POC and Fe_o_/Al_o_ were smaller than those between MAOC/POC and Fe_d_/Al_d_, indicating a stronger positive correlation between amorphous Fe/Al oxides and both SOC fractions. In contrast, pH and Ca_ex_ exhibited a negative influence on both MAOC and POC (Figure 7). Furthermore, VC and ST emerged as key environmental drivers promoting MAOC accumulation: the first two RDA axes explained 20.3 and 40.7% of the total variation, respectively (Figures 7a,c). Similarly, POC showed positive correlations with both VC and ST; however, the influence of ST weakened with increasing soil depth. The proportion of variance explained by the first RDA axis was 25.1% in the surface layer and 45.4% in the subsurface layer (Figures 7b,d).

## 4 Discussion

### 4.1 The divergent vertical responses of MAOC and POC to grassland desertification

Owing to its long-term stability in soil, MAOC has garnered greater attention from researchers than POC has in efforts to increase soil carbon sequestration (Cotrufo and Lavallee, 2022). The data from this study indeed demonstrate that the concentrations of MAOC in both the surface and subsurface layers of the DS were synchronously reduced, whereas a significant decrease in POC was observed only in the surface layer of the DS (Figure 2). These results indicate that different forms of SOC exhibited distinct responses along the vertical soil profile in response to grassland desertification. Existing evidence suggests that POC dynamics depend primarily on carbon inputs from vegetation (Witzgall et al., 2021; Liu et al., 2022; Tang et al., 2023), whereas MAOC formation is driven primarily by the accumulation of microbial necromass (King et al., 2024; Li et al., 2024; Mao et al., 2024), even its ultimate source remains the input of plant carbon. The aboveground biomass of the TS reached 193.99 g/m^2^, which was significantly greater than that of the DS (71.06 g/m^2^) in Inner Mongolia (Peng and Wang, 2016). Our research also revealed that the VC of the TS was significantly greater than that of the DS (Supplementary Table 3). Greater aboveground biomass contributes substantial amounts of litter input to the surface soil with priority. RDA analysis further revealed a stronger positive correlation between VC and both MAOC and POC in the 0–10 cm and 10–20 cm soil layers (Figure 7).

Microbial necromass carbon (MNC) is a crucial source of MAOC, including fungal necromass carbon (FNC) and bacterial necromass carbon (BNC) (Deng and Liang, 2022). Griepentrog et al. (2014) reported that newly formed FNC was 2.6–4.5 times greater than that of BNC in forest soil fractions. Chen et al. (2025) found that the FNC content in MAOC was consistently higher than that in BNC in grassland soils. This disparity can be attributed to the superior microbial utilization efficiency and higher carbon-to-nitrogen ratios of fungi. In this study, higher fungal abundances were detected in both soil layers of the TS (Figure 6c), which indicated that more fungi were involved in the conversion of fresh carbon into stable MAOC in the TS soil and suggested greater production of FNC. Soil microorganisms cannot only directly assimilate dissolved organic compounds released by plant roots and litter but also facilitate the formation of MAOC through the decomposition of POC in soil (Rodrigues et al., 2022). Excessive accumulation of fungi in the subsurface soil of the TS can transform some POC into MAOC, thereby increasing the MAOC content while simultaneously reducing the POC concentration in this soil layer.

### 4.2 The distribution patterns of MAOC in the TS and DS were more closely related to those of Fe_o_/Al_o_

It is widely recognized that higher concentrations of metal elements such as iron and aluminum in minerals are more conducive to the sequestration of SOC (Kleber et al., 2015; Han et al., 2016; Faust et al., 2021). The data herein clearly indicate that the concentrations of Fe/Al oxides in fine soil fractions were higher than those in coarse particles (Figure 4), which was consistent with the observed distribution of SOC in these two distinct soil pools (Figure 2). Relative to Fe_d_/Al_d_, Fe_o_/Al_o_ exhibited a stronger positive correlation with MAOC/POC, as confirmed by RDA analysis (Figure 7). The strong adsorption of Fe/Al oxides primarily stems from two factors: abundant hydroxyl groups and micropores on their surfaces (Kaiser and Guggenberger, 2003; Lalonde et al., 2012). Compared with Fe_d_, Fe_o_ has a larger specific surface area and higher densities of hydroxyl groups, thereby substantially contributing to MAOC retention (Hou et al., 2007; Wang et al., 2016). In this study, the concentration of Fe_o_/Al_o_—specifically those associated with fine particles, rather than Fe_d_/Al_d_—exhibited a substantial difference between the DS and TS. The limited availability of Fe_o_/Al_o_ in the DS significantly restricted their capacity to stabilize MAOC. A lower soil pH enhances the adsorption of MAOC by Fe/Al oxides by increasing the solubility and positive surface charge of these metal oxides (Porras et al., 2017). Thus, the higher pH in the DS (Supplementary Table 3) limited the capacity of these oxides to adsorb organic carbon on their surfaces and exhibited a negative correlation with MAOC accumulation across the TS and DS (Figures 7a,c).

There are several mechanisms through which Ca_ex_ interacts with SOC: Ca_ex_ can form cationic bridges with negatively charged functional groups of organic matter (von Lützow et al., 2007). Ca_ex_ may link two negatively charged organic functional groups as an intermediary (von Lützow et al., 2006). Additionally, Ca_ex_ participates in the formation of ternary complexes involving Fe/Al oxides along with SOC (Sowers et al., 2018). All these functions of Ca_ex_ can only be carried out effectively depending on the input of organic matter into the soil from aboveground plants. Even though the Ca_ex_ content in the DS was higher than that in the TS, the insufficient supply of organic matter limited the accumulation of SOC in the DS.

### 4.3 Lower fungal abundance impairs the storage of MAOC in the DS

In desert steppe ecosystems, soil microbial community composition is shaped by plant presence, with both bacterial and fungal communities being recruited to the rhizosphere and sustained by root exudates (Meier et al., 2017, Yang et al., 2023). However, the infertile and drought soils of desert steppes greatly constrain the availability of plant-derived organic matter for microbial growth. In this study, the fungal abundances in the DS were found to be lower than those in the TS across both soil layers (Figure 6c). Compared with bacteria, fungi produce more chemically recalcitrant structural compounds and have greater carbon use efficiency than bacteria (Jackson et al., 2017; Frey, 2019). The reduction in fungal abundance in the DS was driven primarily by decreases in *Dothideomycetes*, *Eurotiomycetes*, *Sordariomycetes*, and *Geoglossomycetes* (Figure 6c). *Dothideomycetes*, *Eurotiomycetes*, and *Sordariomycetes* were the dominant microbial predictors of SOC storage (Ren et al., 2018; Zhang et al., 2024). *Eurotiomycetes* can produce extracellular cellulases on the basis of fungal cellobiohydrolase gene characterization (Fan et al., 2012). *Sordariomycetes* can decompose organic residues in soils through the excretion of carboxylases and amidolyases (Strope et al., 2011). The lower abundance of these fungi undoubtedly restricted the conversion of organic matter into MAOC in the DS. In addition, the abundance of *Verrucomicrobiota*, one of the dominant bacterial phyla in soil, was significantly lower in the DS than in the TS (Figure 6d). Certain members of *Verrucomicrobiota* can thrive in the rhizosphere and effectively utilize nutrients from root exudates (Da Rocha et al., 2010). These microorganisms can produce hydrolytic enzymes—such as cellulases, xylanases, and chitinases—that enable the degradation of complex polysaccharides (Chen et al., 2017; Naziêbło et al., 2026). Deficiencies in *Verrucomicrobiota* in the DS would restrict their contribution to soil organic carbon storage derived from plant roots.

## 5 Conclusion

**FIGURE 8:**
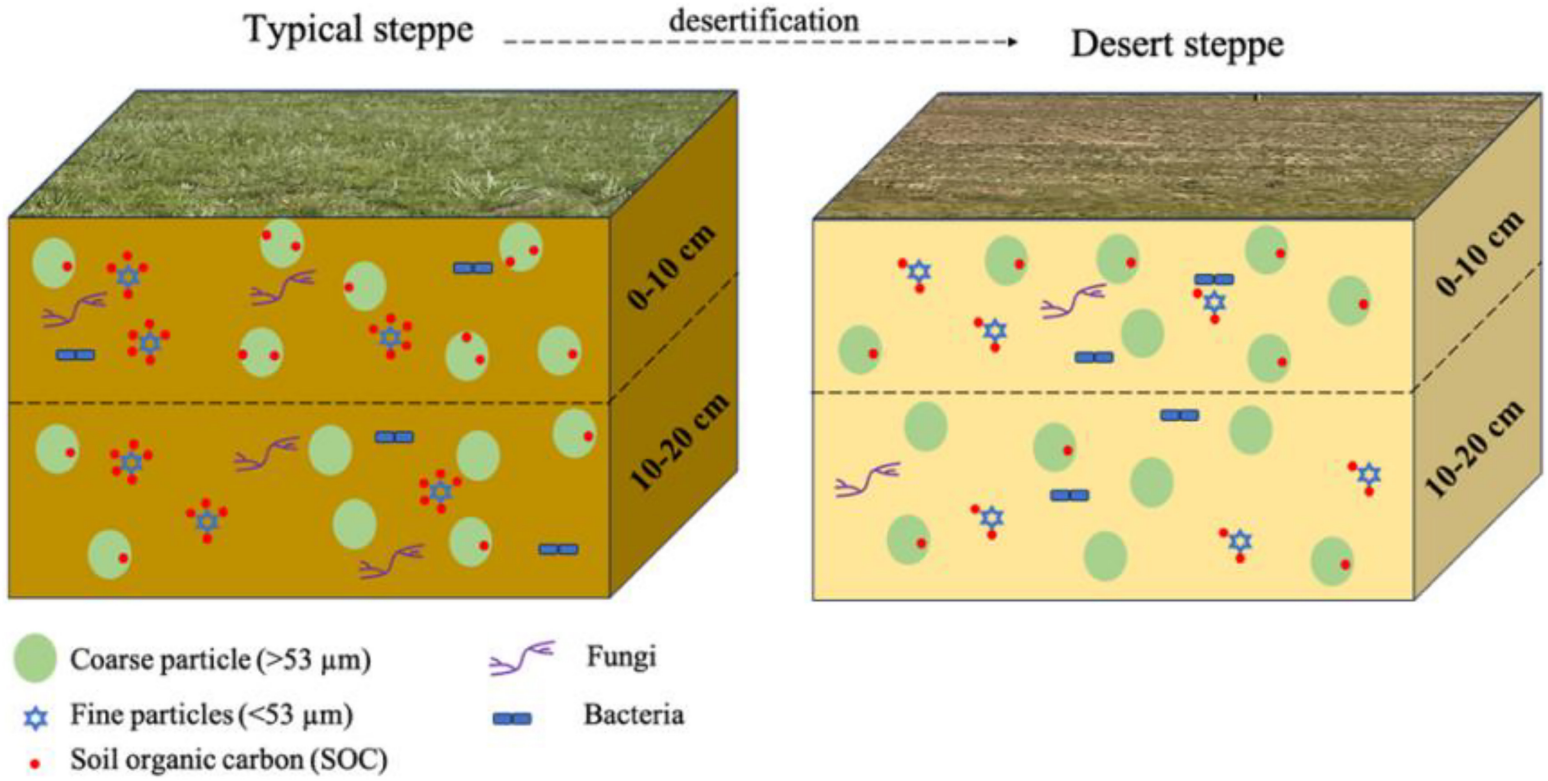
Conceptual picture of differential responses of SOC associated with coarse or fine soil particles along with grassland desertification.

In summary, the decrease in SOC in the DS soil was primarily attributed to low vegetation productivity, compounded by reduced concentrations of Fe_o_/Al_o_ and a lower fungal abundance, which collectively restrict organic matter inputs to the soil and impede the formation of stable mineral-associated carbon (Figure 8). The DS of Inner Mongolia covers an area of approximately 112,000 km^2^, accounting for 10.7% of the total grassland area in the region, which resulted from overgrazing in recent decades (Wang and Zhu, 2001). Appropriate management strategies—such as optimized grazing intensities, multitrophic rewilding, or the restoration of plant diversity—can alter the quality of organic matter inputs and modify environmental conditions in grassland ecosystems, thereby promoting the formation of MAOC and POC.

## Data Availability

The raw data presented in the study are deposited in the NCBI SRA, accession number PRJNA1454534.

## Footnotes

1
https://data.cma.cn
