# Supplementary material for: Differential responses of mineral-associated organic carbon and particulate organic carbon upon desertification of the Inner Mongolian grassland
Source: Front Microbiol. 2026 May 7;17:1814844. doi: 10.3389/fmicb.2026.1814844 (PMC13190598; doi:10.3389/fmicb.2026.1814844)
Supplement: Supplementary file 1 [file Table_1.docx]

Supplementary Material

**Supplementary Table S1. The locations of the sampling points.**

| Sampling point | Desert steppe | Sampling point | Typical steppe |
| --- | --- | --- | --- |
| 1 | 111°13′09.1″ E, 41°13′39.3″ N | 1 | 119°10′16.8″ E, 47°45′37.7″ N |
| 2 | 111°10′35.4″ E, 41°21′30.4″ N | 2 | 119°01′33.7″ E, 47°48′51.8″ N |
| 3 | 111°03′22.8″ E, 41°22′04.7″ N | 3 | 118°56′20.9″ E, 47°50′39.6″ N |
| 4 | 110°57′55.7″ E, 41°23′37.0″ N | 4 | 118°43′23.4″ E, 47°54′46.0″ N |
| 5 | 110°49′49.4″ E, 41°26′47.8″ N | 5 | 118°37′35.3″ E, 47°58′37.6″ N |
| 6 | 110°39′45.7″ E, 41°31′51.1″ N | 6 | 118°30′17.3″ E, 48°02′00.6″ N |
| 7 | 110°35′06.9″ E, 41°35′21.2″ N | 7 | 118°25′26.8″ E, 48°06′26.5″ N |
| 8 | 110°32′10.0″ E, 41°40′12.1″ N | 8 | 118°04′01.3″ E, 48°16′58.0″ N |
| 9 | 110°43′53.8″ E, 41°38′46.1″ N | 9 | 117°49′54.3″ E, 48°21′22.3″ N |
| 10 | 110°41′55.9″ E, 41°38′59.9″ N | 10 | 117°36′22.1″ E, 48°24′19.9″ N |
| 11 | 110°46′05.1″ E, 41°38′50.5″ N | 11 | 117°21′47.5″ E, 48°26′52.7″ N |
| 12 | 110°52′36.7″ E, 41°38′15.9″ N | 12 | 117°14′18.3″ E, 48°28′10.3″ N |
| 13 | 111°06′04.3″ E, 41°38′34.4″ N | 13 | 117°02′30.9″ E, 48°32′06.6″N |
| 14 | 111°19′0.5″ E, 41°38′25.9″ N | 14 | 116°55′12.9″ E, 48°36′46.3″ N |
| 15 | 111°26′33.9″ E, 41°39′09.0″ N | 15 | 116°50′28.9″ E, 48°44′01.0″ N |
| 16 | 111°32′32.2″ E, 41°36′49.6″ N | 16 | 116°53′43.7″ E, 48°51′07.4″ N |
| 17 | 111°35′12.1″ E, 41°34′57.1″ N | 17 | 117°00′02.0″ E, 49°00′48.8″ N |
| 18 | 111°47′30.2″ E, 41°40′36.8″ N | 18 | 117°00′02.4″ E, 49°11′26.4″ N |
| 19 | 112°00′20.6″ E, 41°58′17.9″ N | 19 | 117°04′44.0″ E, 49°19′10.7″ N |
| 20 | 112°11′48.7″ E, 42°70′23.7″ N | 20 | 116°56′08.9″ E, 48°36′02.5″ N |
| 21 | 112°15′56.4″ E, 42°17′01.9″ N | 21 | 118°00′16.1″ E, 49°18′08.8″ N |
| 22 | 112°12′50.7″ E, 42°24′50.9″ N | 22 | 118°10′11.3″ E, 49°06′52.5″ N |
| 23 | 112°17′18.3″ E, 42°29′38.5″ N | 23 | 118°09′58.0″ E, 48°56′58.6″ N |
| 24 | 112°25′10.2″ E, 42°33′42.2″ N | 24 | 117°56′36.0″ E, 48°39′05.3″ N |
| 25 | 112°32′37.5 E″, 42°36′11.6″ N | 25 | 118°00′05.5″ E, 48°43′03.1″ N |

**Supplementary Table S2.** **The microbial composition in the soils of DS and TS (%).**

| Layer  (cm) | Steppe | Archaea | Bacteria | Fungi  (10^-2^) | Metazoa  (10^-3^) | Protoctista  (10^-4^) | Viridiplantae  (10^-2^) | Viruses  (10^-4^) |
| --- | --- | --- | --- | --- | --- | --- | --- | --- |
| 0-10 | DS | 1.10±0.36 | 98.82±0.35 | 1.46±0.26 | 6.50±0.97 | 3.30±2.51 | 6.14±3.41 | 3.25±0.89 |
|  | TS | 1.18±0.40 | 98.69±0.40 | 2.00±1.31 | 6.39±3.80 | 2.58±1.94 | 9.70±5.30 | 3.51±1.90 |
| 10-20 | DS | 1.60±0.80 | 98.31±0.75 | 1.29±0.22 | 4.27±0.32 | 2.02±1.60 | 7.98±9.43 | 3.65±1.52 |
|  | TS | 2.34±1.74 | 97.59±1.73 | 2.15±1.92 | 4.10±1.20 | 4.10±3.90 | 4.95±2.92 | 4.78±2.84 |

Note: The values shown are the means ± SD. DS: desert steppe; TS: typical steppe.

**Supplementary Table S3.** **The environmental parameters of the sampling sites.**

| Environment factor | Typical steppe | | Desert steppe | |
| --- | --- | --- | --- | --- |
|  | 0-10 cm | 10-20 cm | 0-10 cm | 10-20 cm |
| SM (%) | 6.75 ± 1.93 ^a^ | 7.72 ± 2.27 ^a^ | 6.25 ± 1.77 ^b^ | 6.69 ± 2.19 ^b^ |
| ST (°C) | 30.47 ± 1.25 ^a^ | 30.58 ± 1.04 ^a^ | 28.87 ± 1.50 ^b^ | 28.11 ± 1.23 ^b^ |
| pH | 7.80 ± 0.55 ^a^ | 7.98 ± 0.66 ^a^ | 8.61 ± 0.15 ^b^ | 8.66 ± 0.18 ^b^ |
| VC (%) | 69.20 ± 12.05 ^a^ | | 37.04 ± 13.92 ^b^ | |

Note: The values shown are the means ± SD. Different lowercase letters indicate significant differences in different grassland types (P < 0.05 or 0.01). SM: soil moisture; ST: soil temperature; VC: vegetation coverage.
